# Examining the role of intrinsic and extrinsic cues from service requirement narratives in web-based time banking participation decisions

**DOI:** 10.3389/fpubh.2024.1502079

**Published:** 2024-12-13

**Authors:** Huifang Jiao, Meiyan Lin, Lijun Ma, Mei He, Shiguan Yu

**Affiliations:** ^1^School of Management, Shenzhen Institute of Information Technology, Shenzhen, China; ^2^College of Management and Institute of Big Data, Intelligent Management and Decision, Shenzhen University, Shenzhen, China

**Keywords:** time banking, language cues, grounded theory, intrinsic motivation, extrinsic motivation

## Abstract

**Introduction:**

Time banking, known as “Community/Neighborhood Pension,” instantiates a form of co-creation that can provide a new solution to fulfil the unmet social service needs of community members with idle resources, which is a feasible solution to alleviate pension pressure. The sustainable operation of time banks relies on the co-creation and active participation of community members. Therefore, in this study, we investigate the motivation of members to participate in web-based time banks from a service requirement narrative perspective.

**Methods:**

We collected data of 21969 service requirement projects from publicly available information on the website of Nansha Timebank (nstimebank.com, a web-based time bank platform in China). Using the data, we built a model to assess how the intrinsic and extrinsic cues underlying service requirement narratives affect the time bank participation decisions of service providers drawing on grounded theory. Then we conducted a regression analysis to test our hypotheses.

**Results:**

We find that participants respond positively to time coins return and narratives highlighting social connection and value fulfilment but respond negatively to service hour costs and empathy-altruism cues.

**Discussion:**

Our findings suggest that people who receive services in web-based time banking platform should utilize different linguistic cues in service requirement descriptions to improve service exchange results.

## 1 Introduction

The rapid and large-scale growth of global aging is one of the world’s greatest unmet needs. According to the World Social Report 2023, the population of persons 65 years and older in many regions will double in 2050. This issue is also becoming increasingly serious in China and has drawn much interest from scholars and practitioners (1, 2). There were 280.04 million of persons 60 years and older, accounting for 19.8% of the total population in 2022 (3). The proportion of persons 65 years and older is predicted to reach approximately 34% in 2050. Even if there is an increasing need for social care of older people, the current system for meeting that demand is overburdened by the lack of facilities and their subpar quality. It is now critical to determine how to innovate the paradigm for social care of older people (4).

Time banking has emerged as an attractive complement to traditional means of social care for older people, which has been practiced in many countries (3). A time bank is a community-based exchange network where social services are exchanged for time rather than money (5). Community members use a shared space, usually through websites or mobile time banking platforms, to post their service offers and requests; then, they fulfill their service transactions face-to-face offline (6). Traditional social care services social care for older people are usually dominated by brick-and-mortar institutions, and web-based time banking platforms have greatly increased the number of potential service providers and recipients in the community by offering community members the chance to share idle resources (social services). Time banking as a sharing economy model is a sustainable approach and a reliable resource option to solve the deficit in service needs in society by increasing social inclusion and social capital (5). There are idle service resources waiting to be exploited in a community. For example, a time banking member provides services (such as an hour of tutoring) in his spare time and exchanges for time coins that are then exchanged for receiving services (such as an hour of yard work) from another member at a later time (7).

Despite the importance of time banking, scholarly knowledge about this topic is lacking, and the participation rate is relatively low (5). A thorough theoretical foundation is needed for the ongoing study of willingness to engage in time banking. Researchers have noted the vital role of user motivations in influencing participation decisions in sharing economy platforms such as prosocial lending and crowdfunding (8, 9). Time banking is a sharing economy model with traditional service exchange and a field of prosocial giving (10). Service providers evaluate both economic criteria, such as cost and benefit; and prosocial criteria, such as helping others and value fulfillment. Previous studies have focused primarily on diverse factors, such as different motivations (11–14), demographic characteristics (1, 15–17), members’ social goals (18, 19), perceived values (5), social capital (4, 20, 21), and technological features (3, 10, 22), and their influence on the intention to participate. Qualitative data and questionnaire data were used by those studies. However, in web-based time banks, central to service request solicitation is the project narrative, which describes detailed information about the service needed, time coins returned, service hours requested and other personal details. Previous studies have proposed that narratives written by project initiators affect prosocial behaviors in sharing economy models such as prosocial lending and crowdfunding (8, 9). While these studies have advanced our understanding of the role played by project narratives in crowdfunding, we know relatively little about whether, or how, the content of time banking service requirement narratives influences the decisions of service providers.

To address this gap, we assess how service providers respond to both intrinsic and extrinsic cues embedded within service requirement narratives. In summary, this research aims to answer the following questions. (1) Why do service providers choose to engage in time banking? (2) How does the language contained within service requirement narratives convey intrinsic and extrinsic cues to service providers? (3) How do intrinsic and extrinsic motivational appeals affect service providers’ decisions? To answer the research questions, we focused on a web-based time bank, the NanSha Time Bank in China, and collected 21,969 service requirement projects from the website (nstimebank.com). Intrinsic and extrinsic language cues were extracted from the service requirement narratives, and a theoretical model of the influencing factors of service providers’ participation in time banking platforms was developed on the basis of grounded theory. Finally, the model was tested and verified via regression analysis.

This research contributes to the literature and time banking managerial practice in several ways. First, we developed a theoretical model based on grounded theory to explain how the language contained within service requirement narratives conveys extrinsic and intrinsic cues to service providers. We provide a means for researchers to assess how the presence of cues in service requirement narratives may stimulate intrinsic motivation. Second, we verify the effects of different intrinsic and extrinsic cues on service providers’ decision behavior. Our findings suggest that people who receive services should utilize different linguistic cues in service requirement descriptions to improve service exchange results.

## 2 Research background

### 2.1 Community mutual assistance through web-based time banking

Time banking was first proposed by Edgar S. Cahn, who believed that the value of labor could be measured in terms of time and that any labor requiring the same amount of time was equivalent (23). Currently, the time bank is developed into a community-based exchange platform where the value of services is recorded as time coins. The value of labor time is egalitarian priced in a time banking platform regardless of the nature of the service; for example, 1-h house cleaning is valued the same as 1-h tutoring, both worth a time coin (24). Members earn time coins by providing services, deposit them into the time bank, and then spend time coins in exchange for other services (25). Time banking encourages people to join in value creation activities by using their time and skills to help others, which helps them build social capital regardless of their professional or income level. Moreover, people of different ages can benefit from time banking; for example, younger members provide services to deposit time coins and use them to exchange for services when needed (3). Thus, community mutual assistance for social care of older people can be achieved through time banking.

Given the social and practical importance of time banking in the fight to reduce endowment pressure, scholarly examination of time banking has recently begun to flourish. Given the relatively low degree of participation in time banking, researchers have started to explore factors that motivate members to participate. Past time-banking studies shows scholarly focus on several factors that affect time banking participation, including the demographic characteristics, motivations, values and goals, social capital and technological characteristics of the platforms. However, few studies have sought to examine factors that may cause service requests to be more or less attractive to service providers within the time banking context, particularly in terms of service providers that provide services through web-based time banking platforms.

Web-based time banking intermediaries that utilize time-banking platforms have become increasingly popular. Unlike formal brick-and-mortar mutual aid institutions, web-based time-banking platforms facilitate more efficient interactions for members and reduce the work of coordinators (25). There are a growing number of time banking platforms that provide services to members; thus, investigating how information displayed on a website can influence member participation is important for sustainable development of time banking.

### 2.2 Extrinsic and intrinsic factors influencing time banking participation

The time banking participation decision is a hybrid decision form. Time banking participation incorporates aspects of both traditional economic decision-making and psychological factors that influence prosocial giving decisions. According to self-determination theory, human behavior are driven by both extrinsic and intrinsic motivations. Intrinsic motivation arises from within the individual and is driven by a desire for personal satisfaction and enjoyment; extrinsic motivation is driven by external factors such as rewards and recognition (26, 27). Service providers weigh both the extrinsic factors regarding traditional economic behaviors (time coins return and service hours cost) and the intrinsic factors regarding prosocial decisions (help others, feel good about oneself, etc.). Both extrinsic and intrinsic motivations are crucial determinants of prosocial and mutual aid behavior (26).

Time banking is a model of the sharing economy in which service recipients and providers exchange service resources on the platform (6). Time banking began as a means to leverage untapped community capacity to fulfill the unmet service needs of its members (5). Alternatively, individuals are motivated to provide services to needy members in hopes of receiving a financial return on their work. Specifically, service requirement projects offer time coins return to service providers and provide service cost, time and labor. Thus, providing service may be framed as an extrinsic reward, time coins gain. External rewards such as time coins return can increase the likelihood of desired behaviors.

Researchers have also suggested that nonfinancial motivators can also play an influential role. Prosocial givers are motivated to participate because of the psychological gains (i.e., intrinsic rewards) that are garnered from the process of helping (28). Time banking also focuses on building a better society through community mutual assistance (11). In time banking platforms, the value of labor time is egalitarianly priced, and those with highly valued labor time (such as nursing care) opt out unless they are highly motivated by idealistic, social, or altruistic incentives. Thus, providing services on a time banking platform incorporates prosocial behaviors. The extent to which members are motivated to provide services may be influenced by the extent to which they perceive their engagement in the activity of time banking to help needy members (29, 30). From the perspective of self-determination theory, this suggests that individuals may seek to align with their personal goals and values, such as helping others, and be intrinsically motivated to participate in time banking.

The literature on cues has demonstrated that the ways in which language is framed can influence motivation; accordingly, we refer to these as extrinsic and intrinsic cues (31). The way in which individual service request presentations are framed varies across people who receive services, and task framing is known to impact motivation (8). Specifically, providing services may be framed in a way that suggests the existence of an extrinsic reward: cost benefit trade-off, or the existence of intrinsic rewards: helping others, self-satisfaction and social recognition. Previous studies have indicated that monetary incentives are crucial community policies that intrigue users’ extrinsic motivation to inspire knowledge output behavior (32). Research on self-determination theory suggests that verbal praise, which enhances internal feelings of satisfaction, tends to increase intrinsic motivation (27). Thus, we intended to evaluate how extrinsic and intrinsic incentives influence service transaction results by analyzing the language cues displayed in service requirement narratives in time banking platforms.

## 3 Methods

### 3.1 Materials

In this study, we focused on members who sought service needs on the web-based time banking platform nstimebank.com. The Nansha Time Bank is a community mutual assistance service project launched by the Nansha District Government in December 2013. As of December 2021, the “Nansha Time Bank” has grown to 105,810 members, with 139,386 online posts and 89,576 completed services. As such, it represents a valuable context for the study of time banking.

The data for our study were derived from publicly available information on the website of the Nansha Timebank (https://www.nstimebank.com/). The platform provides data about service requirement projects, which include the service type needed, time coins return for the service, service hours request, service transaction result of the project and the description and introduction of the project. The transaction result of a project includes two statuses: completed and not completed. The web page of a service requirement project on the platform is shown in Supplementary Figure 1.

### 3.2 Procedure

We wrote a computer program using the Python computer language to extract information on all projects posted on the platform, collecting each service requirement project’ s information between January 2014 and December 2021. We collected information for 21,969 projects from nstimebank.com. The following projects were removed to ensure validity and accuracy: (1) the word count of the project description text was less than 30; (2) projects with the same description information; (3) projects with incomplete information such as time coins return or service hours; and (4) project information not available to the public. Finally, 12,399 projects remained for analysis.

We conducted two studies to explore service providers’ motivations to participate in web-based time banking from the service requirement narrative perspective. First, a theoretical model of the influencing factors of time-banking participation decisions was proposed on the basis of grounded theory. Second, hierarchical multiple regression analysis was conducted to verify the theoretical model. The study of these “best practices” enabled us to understand the effective language that induced service providers’ motivation.

#### 3.2.1 Analysis of the coding process

This part adopted the data coding method of grounded theory to model intrinsic and extrinsic motivational cues from service requirement project narratives. The inductive research process of programmed grounded theory is widely used and easy to perform (33). Coding, as the core of grounded theory, refers to the continuous and repeated comparisons between events and concepts to enable classification, feature formation, and conceptualization of data (34). The coding process of grounded theory can be formally divided into three interlocking coding processes: open coding, axial coding, and selective coding (35). The text data of service requirement project information crawled from nstimebank.com were analyzed via Nvivo12, a qualitative research data analysis software.

Open coding involves breaking the data into blocks and assigning concepts to account for the data’s meaning. After classification, 8 preliminary categories were extracted: time and effort cost, time coin returns, identity information of disadvantaged people, hardship in life, physical diseases, values from embodiment, helping others, and social desire (Table 1). Axial coding involves relating concepts to each other on the basis of the raw data. After conducting an inductive analysis of the 8 preliminary categories, we finally conceptualized and grouped them into 4 main categories: interest language, empathy-altruism language, values language, and social language (Table 2). Selective coding was a process of integrating and refining the theoretical framework. The main category was identified as the classification of intrinsic and extrinsic language cues that affect service providers’ participation decisions. The intrinsic and extrinsic language cues were characterized as economic motivation, empathy-altruism motivation, values motivation, and social motivation. Finally, we conducted a theoretical saturation test by using 10% of the reserved text data that were randomly selected. The results indicated that no new theoretical viewpoints or new main categories were found. Therefore, the theoretical model established in this study is saturated. The results of the analysis were discussed between the authors to reach a consensus and to ensure the credibility of the interpretation. The research method framework of grounded theory is shown in Supplementary Figure 2.

**Table 1:** Open coding results: the analysis of conceptualization and categorization.

Category	Conceptualization	Original statement (preliminary concept)
Time and effort cost	Demonstrate the service time and effort required to fulfill the service demand	A1: It will take approximately 1.5 h to complete the service. A2: The estimated service time is about half an hour. A3: Working hours are 10:00–12:00, Monday to Friday.
Time coin returns	Demonstrate that service rewards for service providers, such as time coins or subsidies	A4: The service offers 12 Time Coins. A5: The Nansha Time Bank Community Chest Fund is now responsible for covering the subsidy.
Information of disadvantaged people	Display identification information such as orphans, widows, older adults, the infirm, and the disabled in difficulty	A6: The service is aimed at senior citizens and particularly vulnerable older adults who live alone. A7: The service targets are community households of low income and enjoying five guarantees.
Hardship in life	Demonstrate low-income family financial situation and difficulties faced	A8: Living alone in a primary environment, frequently going without food, wearing worn-out clothing, and shivering in the cold. A9: They are financially difficult and can only make ends meet with the government’s bare minimum guarantee.
Physical diseases	Demonstrate the poor physical condition of the person in need of the services.	A10: A person with secondary mental disabilities who is a chronic psychotropic drug user, unresponsive, slow-moving, hunchbacked, diabetic, and has frequent foot pain. A11: The client has dialysis 1–2 times per month because of his kidney failure.
Values form embodiment	Demonstrate the value of service, serve to create a better community, serve the public spirit of volunteerism, convey a positive energy, etc.	A12: To better integrate migrant workers into community life and strengthen their feeling of identity, belonging, and pride in their community. A13: To fervently promote the volunteerism spirit of dedication, love, mutual aid, and advancement. A14: To provide for the needs of the older adults and spread the noble virtue of optimistic thinking.
Helping others	Passing on information about the need for help	A15: Hoping some enthusiasts can help. A16: Hoping that someone in her way will help her renew the outpatient appointment. A17: I, Hong xx, have mobility problems and need someone to clean my house.
Social desire	Express requirements of social communication	A18: I often feel pretty bored at home, and I wish someone would join me for a conversation to break up the monotony. A19: I often feel lonely at home and wish someone would talk to me. A20: I am pretty depressed because of my disease and would appreciate some compassionate individuals coming to my home and chatting with me. A21: I’d like to spend time meeting new friends and participating in senior activities with them.

**Table 2:** Axial coding results: main categories formed by axis coding.

Main category	Preliminary category	Demonstration
Interest language	B1 Time and effort cost B2 Time coins return	The economic attributes of service exchange.
Empathy-altruism language	B3 Information of disadvantaged people B4 Hardship in life B5 Physical diseases B6 Helping others	Information on stimulating empathy and help-seeking messages in service requirement narratives.
Values language	B7 Values from embodiment	Information on ideology and values in service requirement narratives.
Social language	B8 Social desire	Information on social needs and social activities in service requirement narratives.

#### 3.2.2 Theoretical model construction

**Figure 1:**
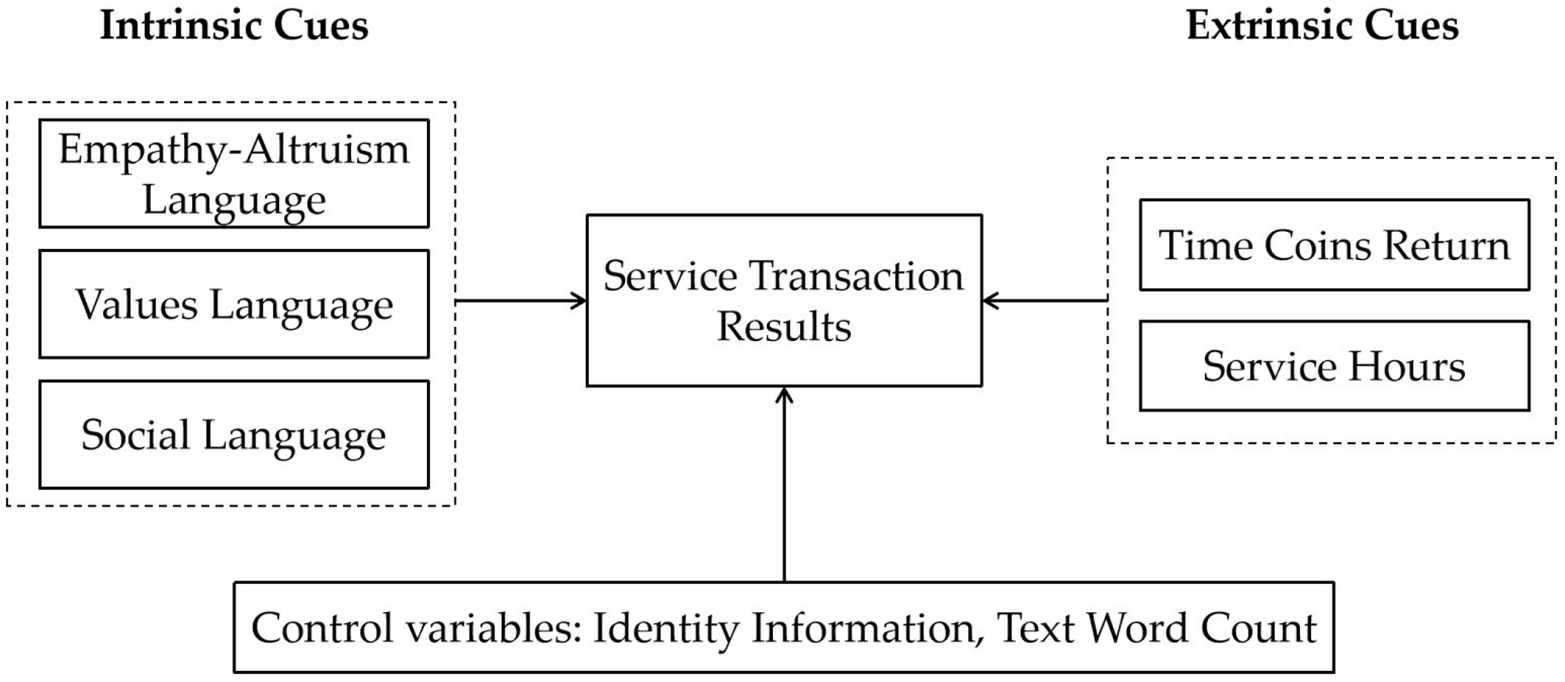
Theoretical model.

On the basis of the analysis and coding of the project information data, we construct a theoretical model of the influencing factors of service providers’ participation decisions on a time banking platform, as shown in Figure 1. The theoretical model stems from a continuous data comparison process that breaks down data into codes, concepts, and categories until theory is saturated. The connotation of the theoretical model of this research mainly includes the following aspects: (1) Service transaction results work as the core category, and other categories influence it; (2) intrinsic language cues and extrinsic language cues, as influencing factors, directly impact service transaction results; (3) the service hours and time coins returns in human interest language are extrinsic motivations, which refer to the service cost and potential reward; and (4) empathy–altruism language, values language and social language are intrinsic motivations, which refer to intangible returns. We draw upon past research in the area of the sharing economy, such as crowdfunding and P2P lending, which suggests that the identity information of the project initiator and the text word count of the description narrative are determinants of funding decisions (30, 36). Thus, the effects of text word count and identity information on service transaction results were controlled.

### 3.3 Hypotheses

Individuals are motivated to provide services to needy members in hopes of receiving a financial return on their work. The way in which individual service request presentations are framed varies across people who receive services, and task framing is known to impact motivation (8). Specifically, providing service may be framed as an extrinsic reward, time coins gain. The literature on cues has demonstrated that the ways in which language is framed can influence motivation; accordingly, we refer to these as extrinsic cues (32). The motivation provided by external rewards can increase the likelihood of desired behaviors. Consumers are rational individuals whose decisions are based on the ability of the behavior to satisfy their utilitarian goals. According to social exchange theory, individuals weigh costs and benefits before engaging in social activities (37). Banking members evaluate the benefit against the cost of providing services to maximize their interests. In the service need project information, service hours and time coin returns can represent the cost and benefit of providing services. Previous studies have also indicated that members engage in time banking for utilitarian goals (5, 11). Thus, when a service need project provides more time coins return and costs fewer service hours, it is likely to be more appealing to service providers. Formally:

*H1*: More service hours needed to complete the service are associated with a decrease in the attractiveness of the service need project among service providers.

*H2*: More time coins return is associated with an increase in the attractiveness of the service need project among service providers.

Altruism suggests that individuals are motivated to offer help for the purpose of benefiting others. Altruism is one of eight mechanisms affecting people’s motivation to engage in philanthropic behavior (38). Empathetic concern to someone who is in need is an important source of altruistic motivation (9). When people acknowledge others’ needs and difficulties, they feel empathetic and willing to help (39, 40). For example, members may feel empathy for the service recipient by acknowledging how difficult situation he/she is in from the narratives; then, they are more likely to help in time banking (41, 42). Thus, intrinsic cues in the narrative—a greater amount of empathy-altruism language—focus on the information of hardship, disadvantage, disease and helping others is salient to the service providers’ reasons for participating. Formally:

*H3*: Greater degrees of empathy-altruism language are associated with an increase in the attractiveness of the service need project among service providers.

Sociality is the primary driver of individuals’ participation in collective behavioral organizations, which refers to the tendency of individuals to engage in social interactions to promote social cohesion (39). Time banking platforms enable members to mutually exchange useful services among themselves and build social capital, especially when most members are unemployed older adults. Time banking services are useful for alleviating loneliness and depression (20). Previous studies have also indicated that people with social motivations participate in time banking for opportunities to spend time with other people (11). Both providing and receiving services in time banking can improve social value, which refers to self-esteem and status benefits from social interaction, subsequently increasing members’ behavioral intention to participate (5). Thus, we propose that a greater amount of social language, which focuses on the needs of companions, chatting and making friends in the narrative, may increase service providers’ willingness to participate. Formally:

*H4*: Greater degrees of social language are associated with an increase in the attractiveness of the service need project among service providers.

Values refer to an individual’s principles, standards or beliefs, which are among the motivations for why people volunteer (11). Time banking platforms enable members to offer community volunteer services and help others, they participate in time banking to act on their values and create a better society, which increases self-satisfaction. Egoism is an explanation for helping, and the need to feel personally fulfilled is one of the reasons why people offer help (9). Self-determination theory also suggests that people engage in prosocial activities to achieve personal satisfaction (43). Previous studies have suggested that members who are motivated by their values are more willing to give time to the time bank (11, 19). Thus, we propose that the presence of value language, which focuses on personal values, beliefs, contributions, and virtues in the narrative, is associated with improved service transaction results. Formally:

*H5*: Greater degrees of values language are associated with an increase in the attractiveness of the service need project among service providers.

### 3.4 Text analysis

We tested the theoretical model of intrinsic and extrinsic cues in the narrative influencing time banking participation decisions in this part. We examined each project’s descriptions written by people who receive services to study linguistic appeals that can trigger different types of motivations of service providers. First, we segment the text data by removing stop words; then, we extract keywords and construct a seed vocabulary from the processed text dataset on the basis of the extrinsic and intrinsic motivation cues. Since time coins return and service hours cost can be directly converted into quantifiable data for extrinsic motivations, three categories of intrinsic language cues—social, values, and sympathy-altruism—are subject to keyword extraction. We extracted the high-frequency words in the text data (the top 150 high-frequency keywords are shown in the Supplementary Table 1) and then calculated the term frequency-inverse document frequency (TF-IDF) value of each word (the top 20 TF-IDF calculated keywords are shown in the Supplementary Table 2). Five coding staff members were then recruited to annotate the three intrinsic language cues. Finally, the initial keywords for the classification of intrinsic language cues are shown in Table 3. Second, we expand the initial keywords by using the Chinese synonym toolkit Synonyms in Python to form an extended vocabulary (Supplementary Table 3). High-frequency keywords in the expanded vocabulary have been included in the expanded keywords for intrinsic language cues, as shown in Table 4.

**Table 3:** Initial keywords for intrinsic language cues.

Variables	Keywords
Social language	Chatting, relief, loneliness, boredom, depression, friendship, visitation, belonging, recreation, companionship.
Empathy-altruism language	Hardship, distress, illness, hardship, pain, help, low income, disadvantage, weakness.
Values language	Volunteer, public welfare, charity, community service, Lei Feng, model, virtue, social worker, respect for older adults, enthusiastic person.

**Table 4:** Expanded keywords for intrinsic language cues.

Variables	Expanded keywords
Social language	Chat, relieve boredom, loneliness, boredom, depression, friendship, visit, accompany, sense of belonging, recreation loneliness, sadness, grief, sadness, uninteresting, tedious, boring, depression, loneliness, chatting, depression, insomnia, schizophrenia, bipolar, pastime, play, pass the time, take pleasure, self-indulgence, visit, seek, look up, attend, return, escort, call on, accompany, pay a return visit, escort, leading, sport activities, recreational activities, cultural activities, cultural performances, socializing, feeling safe, feeling happy, talking, getting to know, making friends.
Empathy-altruism language	Difficulty, dilemma, disease, hardship, pain, help, assist, low-income, vulnerable, frail, Five Guaranteed People, low-income households, onerous, very difficult, difficulty, trouble, distress, dilemma, straits, poor, burden, poverty, peril, situation, illness, hidden worry, illness, complication, cancer, diabetes, infectious disease, chronic, permanent disability, disability, handicap, mobility, disabled, deaf, handicapped low income, low income, extraordinary hardship, low income, help, on behalf of, please, door-to-door, beg, kindness, help the poor, poor households, needy workers, support the poor, help, mutual aid, concern, bad, paralyzed, in poor condition, paralysis.
Values language	Volunteer, public welfare, charity, community service, Lei Feng, exemplary, virtue, social worker, respect for older adults, enthusiast, volunteer, voluntary service, charity, public welfare activities, public service, public welfare, charity, charities, fundraising, fundraising, community activities, service projects, social work, illuminate, character, traditional virtue, noble, sentiment, virtue, noble sentiment, social worker, positive energy, dedication, exemplary, learning from the lightning bolt, respecting the older adults and loving the children.

#### 3.4.1 Measures

##### 3.4.1.1 Independent variables

The three intrinsic language cue items, social language, empathy-altruism language and values language, were measured on the basis of the word list in Table 4. The word list of social language is used to assess the extent to which a narrative concentrates on social activities. The word list of empathy-altruism language focuses on the people who needs will help; it provides a clear picture of the service recipient. The word list of values language is designed to assess the extent to which a narrative concentrates on the values of volunteering and charity. A project description with a specific category language appears was coded as 1; if a specific category language appears multiple times in the project narrative, then we take their sum. A project description may have multiple categories of intrinsic language; then, each category was coded as 1. Extrinsic language, including service hours and time coin returns, focuses on how much cost the provider needs to pay and how many time coins he/she would gain through offering service to a specific project. The extrinsic language cue items were measured directly from the project description. Each service requirement project was required to claim service hours needed and time coins return (see Supplementary Figure 1).

##### 3.4.1.2 Control variables

The identity information refers to the name and address of the project initiator in the text. Some studies have shown that the identity information displayed in project descriptions increases users’ willingness to participate by increasing their trust in the project initiator and reducing their perceived risk of participating (32, 44). This factor was controlled for as a dummy variable; projects with identity information were coded as 1, and those without identity information were coded 0. The text word count refers to the total number of text words of a project description. Related research has demonstrated that the richness of information presented in text affects users’ decisions (36). This factor was controlled for as a continuous variable.

##### 3.4.1.3 Dependent variable

Our dependent variable, the service transaction result, operationalizes the attractiveness of the service project to potential service providers by measuring whether the project was completed. The service transaction result of a project was a dummy variable: 0 = not completed; 1 = completed. The service transaction result of each project is displayed on the web page (see Supplementary Figure 1).

#### 3.4.2 Regression modeling

On the basis of the theoretical model and the factors in the research model, an econometric model was developed to estimate the impact of intrinsic and extrinsic language cues on the willingness of time banking service providers to participate via the following equation.


$$Y=\alpha +\beta D_{i}+\gamma Z_{i}+\varepsilon_{i}$$

Y is the service transaction result. β is the regression coefficient, and γ is the control variable coefficient. α is the intercept. $\varepsilon_{i}$ is the regression error term. $D_{i}$ represents the explanatory variables. $Z_{i}$ represents the set of control variables.

## 4 Results

We used binary logistic regression to analyze the relationships among the variables, investigate how the independent and control variables affect the dependent variables, and identify the main motivational factors that influence service providers’ willingness to participate.

Table 5 presents descriptive statistics and correlations for our variables. 76% of the projects were completed, 49% of the projects displayed identity information of the service receiver. The average number of text words of a project description is 84. The average time coins return of the projects is 36.9, and the average service hours needed for a project is 2.9. As for language cues, empathy-altruism language appears an average of 1.93 times in all projects, the numbers for social and values language are 0.7 and 0.67, respectively. The absolute values of the correlation coefficients among the variables are less than 0.8 indicating that multicollinearity was not a threat. The coefficients are all significant at *p* < 0.001 or *p* < 0.01 level demonstrating statistical significance.

**Table 5:** Correlations, means, and standard deviations (SDs).

	Variables	Mean	SD	1	2	3	4	5	6	7
1	Service transaction results	0.76	0.43							
2	Identity information	0.49	0.51	0.088						
3	Text word count	84.00	30.24	−0.147	−0.150					
4	Time coins return	36.90	45.40	0.104	0.126	−0.22				
5	Service hours	2.90	6.85	−0.098	0.103	−0.184	0.736			
6	Empathy-altruism language	1.93	2.17	−0.041	0.105	0.092	−0.119	−0.117		
7	Social language	0.70	1.13	0.069	0.079	0.064	−0.120	−0.100	−0.031	
8	Values language	0.67	0.47	0.049	−0.040	0.062	0.105	0.111	0.045	−0.072

*N = 12,399. Correlations that exceed |0.01| are significant at p < 0.01.*|N = 12,399. Correlations that exceed |0.01| are significant at p < 0.01.|N = 12,399. Correlations that exceed |0.01| are significant at p < 0.01.

Table 6 presents the results of our regression analysis. All control variables were entered into Model 1. The two measures of extrinsic cues (time coins return and service hours) were entered into Model 2. Model 3 adds the composite measures of intrinsic cues (empathy-altruism language, social language, values language). Inclusion of control variables in model 1 accounted for a proportion of the variance (Nagelkerke *R*^2^ = 0.073) in service transaction results. Inclusion of extrinsic and intrinsic language cues in Model 3 significantly increased variance explained (Nagelkerke *R*^2^ = 0.212), which indicates that the explanatory power of our model is acceptable. The beta weights revealed significant effects of extrinsic language cues on service transaction results. Time coins return (*β* = 0.453, *p* < 0.001) and service hours (*β* = -0.107, *p* < 0.001) predicted service transaction results, thus Hypothesis 1 and Hypothesis 2 were supported. As for intrinsic language cues, social language (*β* = 0.500, *p* < 0.001) and values language (*β* = 0.189, *p* < 0.05) have significant positive effects on service transaction results, which provide support for Hypothesis 4 and Hypothesis 5. However, empathy-altruism language is negatively related to service transaction results (*β* = -0.203, *p* < 0.05), which is contrary to Hypothesis 3.

**Table 6:** Table of regression analysis results.

Variables	Model 1	Model 2	Model 3
Identity information	0.270*** (0.082)	0.291*** (0.082)	0.290*** (0.084)
Text word count	−0.828*** (0.067)	−0.89*** (0.129)	−1.249*** (0.132)
Time coins return		0.560*** (0.092)	0.453*** (0.093)
Service hours		−0.114*** (0.025)	−0.107*** (0.025)
Empathy-altruism language			−0.203* (0.100)
Social language			0.500*** (0.088)
Values language			0.189* (0.090)
Constants	4.626*** (0.304)	8.245*** (0.773)	7.541*** (0.773)
Nagelkerke *R*^2^	0.073	0.135	0.212
*N*	12,399	12,399	12,399

***p < 0.001; **p < 0.01; *p < 0.05; Standard errors in parentheses (#.##).

## 5 Discussion

In terms of extrinsic language cues, time coins return is positively related to service completion, and service hours is negatively related to service completion. The results indicate that greater service hours needed to complete the task would be associated with less willingness for service providers to participate; thus, the probability of the project being completed is lower. Further, more time coins return would be associated with an increase in the willingness of service providers to participate; thus, the transaction result of the project is more likely to be completed. The results confirm that extrinsic motives embedded in project description narratives have significant effects on rational service providers’ decision-making behaviors when they pursue utilitarian goals.

With respect to intrinsic language cues, evidences are provided for positive effects of social language and values language. The results indicated that greater degrees of social language and values language would be associated with an increase in the probability of a project being completed. As expected, we found that intrinsic motives (social and values language) embedded in the project description narratives significantly increased service providers’ willingness to participate. However, empathy-altruism language is negatively related to service completion. Contrary to our expectation, increasing the focus on empathy-altruism motives embedded in the service request narratives significantly diminished service providers’ interest in the project. This result might be explained by the affective load theory (45). ALT recognizes that negative emotions such as anxiety, boredom, and frustration can increase the affective load, thereby inducing avoiding, resisting and giving up behaviors (46, 47).

Among the control variables, identity information has a positive effect on service completion. Identifying information can increase the credibility of a project; thus, people who receive services should try to include more accurate identity information when posting requirements. The text word count is negatively related to service completion, which indicates that too much text will make the service provider less willing to offer service. According to affective load theory, when users are exposed to too much information, it can lead to mental fatigue and prevent them from engaging in participation behavior (47).

## 6 Conclusion

In this study, we attempt to gain a deeper understanding of whether service provider attraction to service requirement projects is influenced by extrinsic and intrinsic cues embedded in project description narratives. By doing so, we provide the first examination of the role played by different types of cues in time banking platforms and propose a theoretical framework for predicting the participation decisions of service providers. Our findings support those of prior studies by suggesting that extrinsic and intrinsic motivations affect decisions in the time banking context. Given the importance of web-based time banking in community pensions, it is vital to develop an understanding of member participation in these fields.

Consistent with self-determination theory, this study distinguishes extrinsic motivations and extrinsic motivations and proves that both motivations play a role in members’ participation decisions. People assess benefits and costs to maximize their interest in time banking participation decisions; specifically, external rewards increase service providers’ intention to participate, and service hours decrease their intention (48). We also suggest the effects of intrinsic language cues, including social language and values language, in strengthening preexisting intrinsic motivations by using a content analysis methodology. Social and value motivations are the main factors that influence member participation in time banking. Positive information, such as social and value cues included in the service requirement narrative, can encourage providers to participate.

In our analysis, empathy-altruism has a significant negative effect on intrinsic motivation, which is inconsistent with previous findings in the charitable giving literature. Negative information about the service recipient’s hardships, sorrows, and tragedies that intend to induce empathy-altruism motivation does not facilitate service transactions. The empathy-altruism language assesses the extent to which a narrative expresses how sympathetic the service recipient is and how desperately he/she is in need of help. The word list includes dilemmas, diseases, hardship, pain, etc. When people are exposed to overload negative information, negative emotions arise and decrease their intention to offer help.

### 6.1 Implications for practitioners

For practitioners, web-based time banking platforms represent a valuable tool for bolstering community pension activities. Our research provides time banking platforms with managerial implications for how to effectively promote service transaction projects to community members and improve service providers’ interest in participating. Our findings suggest that financial incentives are attractive to service providers. The return of time coins benefits service providers, and it is necessary for time banking platforms to improve the value of time coins. Thus, it is suggested that time banking platforms explore diverse time coin exchange systems, such as exchanges for commodities. It is also suggested that time banking platforms adopt differential pricing for different services and that members providing highly valued labor (such as nursing care) can gain more time coins than less valued labor (such as cleaning); thus, the services provided by time banking can attract more members to participate.

Furthermore, our results suggest that service need projects tend to be completed when their descriptions are framed to appeal to the intrinsic reasons (idealistic and social incentives) that service providers need to participate. In web-based time banking practices, using language cues can stimulate members’ interest and motivation, which seems to be a better way than solely addressing financial rewards. It is suggested that people who receive services use more intrinsic cues that emphasize the role of service providers. To stimulate members to provide services in time banking, the service request project narrative should emphasize service providers’ psychological gains, such as self-value fulfillment and social needs satisfaction. Moreover, our research suggests that intrinsic cues such as the hardship and sorrows of people who receive services are negative information that hinders service providers’ intention. It appears that more empathy-altruism information does not lead to better results; thus, the service requirement narrative should not focus on the language needed to help others too much.

### 6.2 Limitations and future research

In this study, we focus on the effect of language cues on time banking participation decisions. The data of service requirement projects posted by people who receive services were collected from the website of a specific Chinese time bank platform. The results of this research seems to provide insights into China’s time banking landscape, future research could collect data from other time banks and from regions with different social and cultural background. The constructs are measured via computer-aided text analysis, and the application of traditional manual content analysis to time banking narratives may reveal additional constructs for future investigations. For example, future studies may investigate how other extrinsic and intrinsic cues may affect service transaction results. This will provide more insights into the construction of service requirement narratives that maximize the effectiveness of time banking practices. The method of content analysis can also be applied to multimedia data such as photos, and future research can examine how image information included in service requirement projects affects participation decisions. The psychometric mechanism of how intrinsic and extrinsic cues in service requirement narratives influence motivations needs to be explored further. The use of an experimental method to explore how members decide to provide service is suggested. Members in the experiment could be presented with varying types of appeals, their behaviors can be tracked, and their motivation can be directly accessed via psychometric measurements.

## Data Availability

The raw data supporting the conclusions of this article will be made available by the authors, without undue reservation.

## References

[1] Wu Y Ding Y Hu C Wang L. The influencing factors of participation in online Timebank nursing for community elderly in Beijing, China. Front Public Health. (2021) 9:1–14. doi: 10.3389/fpubh.2021.650018, PMID: 33898381 PMC8060504

[2] Wu Z Xu C Zhang L Wang Y Leeson GW Chen G. Volunteering and depression among older adults: an empirical analysis based on CLASS 2018. Int J Ment Health Promot. (2023) 25:403–19. doi: 10.32604/ijmhp.2023.024638

[3] Jiao H Lin M Ma L He M Guo C. Understanding the determinants of member participation intentions in web-based time banking platforms: role of perceived trust and perceived risk. Behav Inform Technol. (2024) 10:1–17. doi: 10.1080/0144929X.2024.2336610

[4] Leung WKS Chang MK Cheung ML Shi S. Swift trust development and prosocial behavior in time banking: a trust transfer and social support theory perspective. Comput Hum Behav. (2022) 129:107137. doi: 10.1016/j.chb.2021.107137

[5] Kakar AK. Investigating factors that promote time banking for sustainable community based socio-economic growth and development. Comput Hum Behav. (2020) 107:105623. doi: 10.1016/j.chb.2018.07.034

[6] Válek L Jašíková V. Time Bank and sustainability: the permaculture approach. Procedia Soc Behav Sci. (2013) 92:986–91. doi: 10.1016/j.sbspro.2013.08.788

[7] Whitham MM Clarke H. Getting is giving: time banking as formalized generalized exchange. Sociol Compass. (2016) 10:87–97. doi: 10.1111/soc4.12343

[8] Allison TH Davis BC Short JC Webb JW. Crowdfunding in a prosocial microlending environment: examining the role of intrinsic versus extrinsic cues. Entrep Theory Pract. (2015) 39:53–73. doi: 10.1111/etap.12108

[9] Zhang H Chen W. Backer motivation in crowdfunding new product ideas: is it about you or is it about me? J Prod Innov Manag. (2019) 36:241–62. doi: 10.1111/jpim.12477

[10] Bellotti V. Cambridge S. Hoy K. Shih P. C. Handalian L. Han K.. (2014), Towards community-centered support for peer-to-peer service exchange: rethinking the timebanking metaphor. Proceedings of ACM conference on human factors in computing systems. New York: ACM. 2975–2984.

[11] Collom E. Motivations and differential participation in a community currency system: the dynamics within a local social movement organization. Sociol Forum. (2011) 26:144–68. doi: 10.1111/j.1573-7861.2010.01228.x

[12] Shih P. C. Bellotti V. Han K. Carroll J. M. Unequal time for unequal value: implications of differing motivations for participation in timebanking. Proceedings of the 33rd annual ACM conference on human factors in computing systems, (2015), New York: ACM. 1075–1084.

[13] Mcguirk E. Timebanking in New Zealand as a prefigurative strategy within a wider degrowth movement. J Polit Ecol. (2017) 24:595–609. doi: 10.2458/v24i1.20897

[14] Dury S. Dynamics in motivations and reasons to quit in a care Bank: a qualitative study in Belgium. Eur J Ageing. (2018) 15:407–16. doi: 10.1007/s10433-017-0455-y, PMID: 30532677 PMC6250642

[15] Collom E. Engagement of the elderly in time banking: the potential for social capital generation in an aging society. J Aging Soc Policy. (2008) 20:414–36. doi: 10.1080/08959420802186282, PMID: 19042555

[16] Lasker J Collom E Bealer T Niclaus E Young Keefe J Kratzer Z. Time banking and health: the role of a community currency organization in enhancing well-being. Health Promot Pract. (2011) 12:102–15. doi: 10.1177/1524839909353022, PMID: 20685912

[17] Carnero MA Martinez B Sánchez-Mangas R. Explaining transactions in time banks in economic crisis. Appl Econ Lett. (2015) 22:739–44. doi: 10.1080/13504851.2014.975323

[18] Valor C Papaoikonomou E. Time banking in Spain exploring their structure, management and users’ profile. Rev Int Sociol. (2016) 74:e028. doi: 10.3989/ris.2016.74.1.028

[19] Valor C Papaoikonomou E Martínez-de-Ibarreta C. Consumer-to-consumer exchanges: a goal theory approach in the time banking context. Span J Mark -ESIC. (2017) 21:14–24. doi: 10.1016/j.sjme.2016.12.002

[20] Seyfang G. Growing cohesive communities one favour at a time: social exclusion, active citizenship and time banks. Int J Urban Reg Res. (2003) 27:699–706. doi: 10.1111/1468-2427.00475

[21] Tina Yuan CW Hanrahan B Carroll JM. Is there social capital in service exchange tools?: investigating timebanking use and social capital development. Comput Hum Behav. (2018) 81:274–81. doi: 10.1016/j.chb.2017.12.029

[22] Yuan CW Hanrahan B Carroll JM. Assessing timebanking use and coordination: implications for service exchange tools. Inf Technol People. (2019) 32:344–63. doi: 10.1108/ITP-09-2017-0311

[23] Cahn E. On lets and time dollars. Int J Community Curr Res. (2001) 5:1–17. doi: 10.15133/j.ijccr.2001.004

[24] Tucnik P Valek L Blecha P Bures V. Use of Timebanking as a non-monetary component in agent-based computational economics models. WSEAS Trans Bus Econ. (2016) 13:229–37.

[25] Han K Shih PC Bellotti V Carroll JM. It's time there was an app for that too: a usability study of mobile timebanking. Int J Mob Hum Comput Interact. (2015) 7:1–22. doi: 10.4018/ijmhci.2015040101

[26] Choy K Schlagwein D. Crowdsourcing for a better world on the relation between IT affordances and donor motivations in charitable crowdfunding. Inf Technol People. (2016) 29:221–47. doi: 10.1108/ITP-09-2014-0215

[27] Ryan RM Deci EL. Intrinsic and extrinsic motivations: classic definitions and new directions. Contemp Educ Psychol. (2000) 25:54–67. doi: 10.1006/ceps.1999.1020, PMID: 10620381

[28] Gagné M Deci EL. Self-determination theory and work motivation. J Organ Behav. (2005) 26:331–62. doi: 10.1002/job.322

[29] Parsons LM. The impact of financial information and voluntary disclosures on contributions to not-for-profit organizations. Behav Res Account. (2007) 19:179–96. doi: 10.2308/bria.2007.19.1.179

[30] Majumdar A Bose I. My words for your pizza: an analysis of persuasive narratives in online crowdfunding. Inf Manag. (2018) 55:781–94. doi: 10.1016/j.im.2018.03.007

[31] Cimpian A Arce H-MC Markman EM Dweck CS. Subtle linguistic cues affect Children’s motivation. Psychol Sci. (2007) 18:314–6. doi: 10.1111/j.1467-9280.2007.01896.x, PMID: 17470255

[32] Guo S Guo X Fang Y Vogel D. How doctors gain social and economic returns in online health-care communities: a professional capital perspective. J Manag Inf Syst. (2017) 34:487–519. doi: 10.1080/07421222.2017.1334480

[33] Corbin J Strauss A. Basics of qualitative research: techniques and procedures for developing grounded theory. New York: Sage Publications (2008).

[34] Mediani H. An introduction to classical grounded theory. SOJ Nurs Health Care. (2017) 3:1–5. doi: 10.15226/2471-6529/3/3/00135

[35] Edwards-Jones A. Qualitative data analysis with NVIVO. J Educ Teach. (2014) 40:193–5. doi: 10.1080/02607476.2013.866724

[36] Bi S Liu Z Usman K. The influence of online information on investing decisions of reward-based crowdfunding. J Bus Res. (2017) 71:10–8. doi: 10.1016/j.jbusres.2016.10.001

[37] Zhao Q Chen C-D Wang J-L Chen P-C. Determinants of backers’ funding intention in crowdfunding: social exchange theory and regulatory focus. Telematics Inform. (2017) 34:370–84. doi: 10.1016/j.tele.2016.06.006

[38] Bekkers R Wiepking P. A literature review of empirical studies of philanthropy: eight mechanisms that drive charitable giving. Nonprofit Volunt Sect Q. (2011) 40:924–73. doi: 10.1177/0899764010380927

[39] Knoke D. Incentives in collective action organizations. Am Sociol Rev. (1988) 53:311–29. doi: 10.2307/2095641

[40] Dovidio JF. The empathy- altruism hypothesis: paradigm and promise. Psychol Inq. (1991) 2:126–8. doi: 10.1207/s15327965pli0202_4

[41] Woolcock MJV. Learning from failures in microfinance. Am J Econ Sociol. (1999) 58:17–42. doi: 10.1111/j.1536-7150.1999.tb03281.x

[42] Herzenstein M Sonenshein S Dholakia UM. Tell me a good story and I may lend you money: the role of narratives in peer-to-peer lending decisions. J Mark Res. (2011) 48:S138–49. doi: 10.1509/jmkr.48.SPL.S138

[43] Deci EL. Effects of externally mediated rewards on intrinsic motivation. J Pers Soc Psychol. (1971) 18:105–15. doi: 10.1037/h0030644

[44] Parhankangas A Renko M. Linguistic style and crowdfunding success among social and commercial entrepreneurs. J Bus Ventur. (2017) 32:215–36. doi: 10.1016/j.jbusvent.2016.11.001

[45] Nahl D. Social–biological information technology: an integrated conceptual framework. J Am Soc Inf Sci Technol. (2007) 58:2021–46. doi: 10.1002/asi.20690

[46] Labrague LJ. Facebook use and adolescents' emotional states of depression, anxiety, and stress. Health Sci J. (2014) 8:80–9.

[47] Thatcher A Vasconcelos AC Ellis D. An investigation into the impact of information behaviour on information failure: the Fukushima Daiichi nuclear power disaster. Int J Inf Manag. (2015) 35:57–63. doi: 10.1016/j.ijinfomgt.2014.10.002

[48] Dovidio JF Piliavin JA Schroeder DA Penner LA. The social psychology of prosocial behavior. New York: Psychology Press (2006). 10.1146/annurev.psych.56.091103.070141 15709940

