# Supplementary material for: Examining the role of intrinsic and extrinsic cues from service requirement narratives in web-based time banking participation decisions
Source: Front Public Health. 2024 Dec 13;12:1502079. doi: 10.3389/fpubh.2024.1502079 (PMC11671481; doi:10.3389/fpubh.2024.1502079)
Supplement: Supplementary file 1 [file Supplementary_file_1.docx]

Supplementary Material

**Supplementary Figure 1** shows a specific service requirement project description page displayed on the official website of the Nansha Time Bank. In general, an individual needs to first register as a member on the official website of the Time Bank and then initiate a service requirement project through the Time Bank platform, including providing detailed information such as the requirement description, service type, time coins payment, service time, and service location. Service providers can browse and read the details of the service requirement narrative through the website of the Nansha Time Bank and then forward or provide services according to their own wishes.


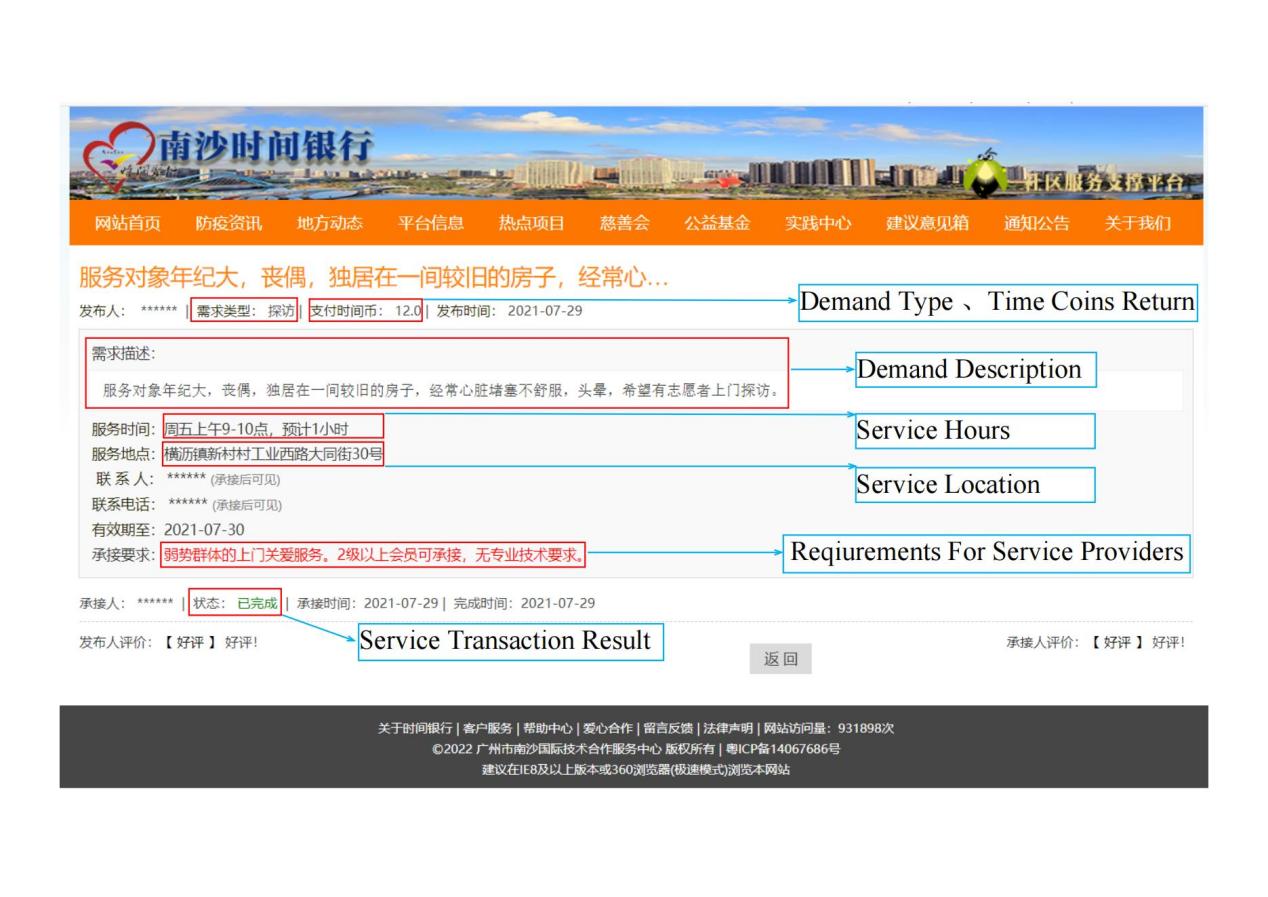


**Supplementary Figure 1.** The web page of a service request project on the Nansha Time Bank


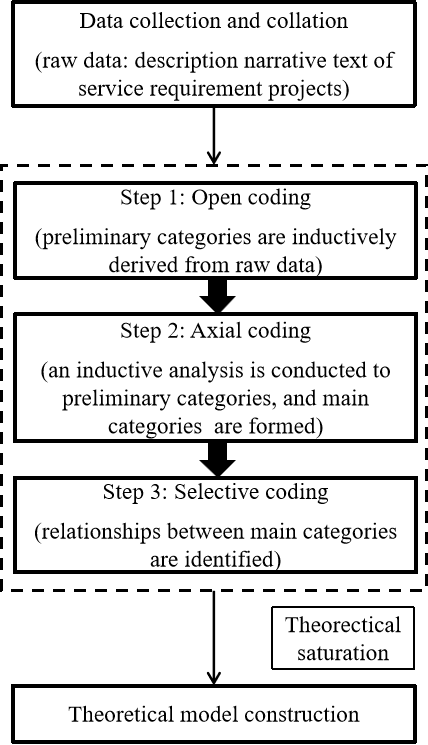


**Supplementary Figure 2.** The research method flow of grounded theory

**Supplementary Table 1.**Top 150 high-frequency keywords

| Words | Freq | Words | Freq | Words | Freq | Words | Freq |
| --- | --- | --- | --- | --- | --- | --- | --- |
| Community | 1944 | Burden | 141 | Friendship | 90 | Condolences | 73 |
| Volunteer | 1909 | Propagate | 136 | Consolation | 90 | Share | 73 |
| Hope | 1512 | Dilemma | 135 | Assistance | 90 | Daily necessities | 60 |
| Volunteers | 1484 | Economy | 134 | Charity | 90 | Talk | 60 |
| Difficulties | 845 | Conditions | 128 | Pioneers | 90 | Lei Feng Spirit | 60 |
| Visit | 698 | Order | 126 | Low-income insurance | 89 | Patriotism | 60 |
| Disability | 582 | Support | 126 | Encourage | 89 | Honor | 60 |
| Chat | 526 | Concern | 123 | Tough | 89 | Respect the aged | 60 |
| Enthusiasm | 495 | Release | 123 | Gratitude | 88 | Public service | 60 |
| People in need | 485 | Low-income household | 114 | Older adults with no family | 86 | The good life | 58 |
| Help | 477 | Pension | 114 | Patriotism | 86 | Vulnerable | 58 |
| Wish | 477 | Volunteer team | 114 | Blind | 86 | Depend on each other | 57 |
| Lighten | 424 | Lonely | 111 | Severely disabled | 86 | Women’s Federation | 57 |
| Enthusiasts | 408 | Diversion | 111 | Model | 86 | Getting things done | 57 |
| Publicity | 370 | Stroke | 111 | Station | 84 | Neighborhood | 55 |
| Older adults | 360 | Lectures | 111 | Neighborhood committee | 84 | Contribution | 55 |
| Mobility problems | 356 | Happiness | 107 | Weakness | 84 | Gratitude | 50 |
| Community services | 318 | Reunion | 107 | People’s livelihood | 84 | Dementia | 50 |
| Live alone | 300 | Service Center | 107 | Crowd | 84 | Incidence | 50 |
| Love | 278 | Voluntary worker | 100 | Grassroots | 83 | Blindness | 50 |
| Thank you | 271 | Make friends | 100 | Send warmth | 83 | Cohesion | 50 |
| Festivals | 267 | Necessities | 100 | Loneliness | 82 | Glorious tradition | 50 |
| Care | 258 | Supplies | 100 | Attribution | 82 | Relieve boredom | 46 |
| Social worker | 246 | Skills | 98 | Illness | 82 | Consolation | 46 |
| Respect for older adults | 222 | Reply on | 98 | Volunteer clinic | 82 | Hardship case | 46 |
| United | 200 | Mutual aid | 98 | Friendship | 82 | Carry out | 46 |
| Warmth | 197 | Activities | 96 | Sense of belonging | 80 | Giving | 46 |
| Low-income insurance | 196 | Boredom | 96 | Kindness | 80 | Depression | 46 |
| Nursing home | 181 | Virtues | 96 | Pain | 80 | Aging | 46 |
| Accompaniment | 178 | Living goods | 95 | Meal delivery | 80 | Stay behind | 46 |
| Public welfare | 173 | Traditional virtues | 94 | Depression | 79 | Assistance | 46 |
| Care | 170 | Psychology | 94 | Good Samaritans | 79 | Ailment | 40 |
| Self-care | 168 | Inherit | 92 | Interdependent | 78 | Happiness | 40 |
| Have a chat | 166 | Promote | 92 | Amateur life | 78 | Aging population | 40 |
| Organization | 157 | Disease | 92 | Exemplary role | 76 | Cultural entertainment | 39 |
| Branch | 154 | Pressure | 92 | Loneliness | 76 | Fun | 39 |
| Donation | 154 | Five guarantees | 91 | Learn from Lei Feng | 76 |  |  |
| Party Branch | 144 | Lei Feng | 90 | Benefit the people | 73 |  |  |

**Supplementary Table 2.** Top 20 keywords based on the TF-IDF calculation

| Serial number | Words | Serial number | Words |
| --- | --- | --- | --- |
| 1 | Volunteer | 11 | Wishful thinking |
| 2 | Community | 12 | Enthusiasm |
| 3 | Volunteers | 13 | Light up |
| 4 | Hope | 14 | Mobility problems |
| 5 | Visiting | 15 | Community services |
| 6 | Disability | 16 | Help |
| 7 | People in need | 17 | Social worker |
| 8 | Difficulties | 18 | Living alone |
| 9 | Enthusiasts | 19 | respect for the aged |
| 10 | Chat | 20 | Meal delivery |

**Supplementary Table 3.** Results of synonym expansion

| High-Frequency Words | Relevance | High-Frequency Words | Relevance |
| --- | --- | --- | --- |
| Alone | 1. Alone: 1.0  2. Loneliness: 0.79520726  3. Lonesome: 0.76940274  4. Sad: 0.702606  5. Miss: 0.7018169  6. Sadness: 0.68276787  7. Misery: 0.6790234  8. Sentimental: 0.6392681  9. Serenity: 0.62291014  10. Apprehension: 0.6150053 | Boredom | 1. Tedium: 1.0  2. Uninteresting: 0.77148384  3. Ennui: 0.7183282  4. Depressed: 0.70235497  5. Dislike: 0.6939903  6. Apathy: 0.68333954  7. Tiredness: 0.6812404  8. Childish: 0.638813  9. Confusion: 0.6337119  10. Laziness: 0.6323888 |
| Depression | 1. Depression: 1.0  2. Upset: 0.8297169  3. Doldrums: 0.81046814  4. Insomnia: 0.76592773  5. Obsessive-compulsive disorder: 0.7624636  6. Schizophrenia: 0.73021406  7. Disease: 0.7185986  8. Epilepsy: 0.71350574  9. Schizophrenia: 0.7048504  10. Bipolar: 0.69336826 | Relieve boredom | 1. Relief of boredom:1.0  2. Pastime: 0.6770339  3. Play: 0.64848065  4. Dispatch: 0.64518976  5. Playing cards: 0.6103776  6. Drinking tea: 0.5761996  7. Gossip: 0.57594293  8. For fun: 0.5735397  9. Self-indulgence: 0.5698817  10. Watch the show: 0.5483093 |
| Visit | 1. Visiting: 1.0  2. Pay a visit: 0.7210812  3. Search: 0.7048898  4. Visitation: 0.70253474  5. Drop by: 0.68218833  6. Interview: 0.63687235  7. Approach: 0.57771415  8. Return visit: 0.55999714  9. Investigate: 0.5544117  10. Revisit: 0.5348015 | Accompaniment | 1. Accompany: 1.0  2. Escort: 0.6461504  3. Visiting: 0.63686055  4. Lead: 0.63423264  5. Accompanying: 0.6289706  6. Meet: 0.62500274  7. Entourage: 0.61616486  8. Worship: 0.61061233  9. Companion: 0.58916366  10. Lead a team: 0.57877094 |
| Sense of Belonging | 1. Sense of Belonging: 1.0  2. Identity: 0.83084154  3. Cohesion: 0.7436758  4. Sense of security: 0.71598697  5. Happiness: 0.70082134  6. Sense of honor: 0.69286746  7. Pride: 0.6881018  8. Sense of trust: 0.63874143  9. Creativity: 0.6340194  10. Sense of superiority: 0.6045321 | Cultural and recreational activities | 1. Cultural and recreational activities: 1.0  2. Cultural and sports activities: 0.80387574  3. Community activities: 0.74572974  4. Recreational activities: 0.69763225  5. Cultural activities: 0.6901878  6. Cultural performance: 0.6687378  7. Large events: 0.6356761  8. Social activities: 0.62897366  9. Holding events: 0.61474115  10. Recreation activities: 0.6128886 |
| Difficulties | 1. Difficulty: 1.0  2. Hardship: 0.6630496  3. Onerous: 0.6418483  4. Difficult: 0.64124155  5. Hard: 0.6305862  6. Trouble: 0.6264315  7. Dilemma: 0.62160414  8. Distress: 0.61141205  9. Tightness: 0.6099264  10. Embarrassment: 0.59724635 | Dilemma | 1. Dilemma: 1.0  2. Quagmire: 0.8684037  3. Predicament: 0.8365001  4. Mud puddle: 0.6856085  5. Quandary: 0.6854408  6. Jeopardy: 0.6732176  7. Situation: 0.6688215  8. Bouts of pain: 0.6684857  9. Hidden worries: 0.66584957  10. Distress: 0.6423848 |
| Disability | 1. Disability: 1.0  2. Damaged: 0.83098507  3. Defeated: 0.817337  4. Intellectual disability: 0.7198358  5. Mobility: 0.699303  6. People with disabilities: 0.66233575  7. Disabiling: 0.6522248  8. Deafness: 0.6306813  9. Handicapped: 0.6099498  10. Physical disability: 0.5718358 | Diseases | 1. Disease: 1.0  2. Illness: 0.8571499  3. Complications: 0.8268104  4. Infectious diseases: 0.80427927  5. Chronic diseases: 0.8010147  6. Cancer: 0.778106  7. Diabetes: 0.7602003  8. The disease: 0.7578845  9. Chronic: 0.7545724  10. Gynecological diseases: 0.7535223 |
| Care | 1. Solicitude: 1.0  2. Poverty alleviation: 0.85433966  3. Love: 0.74287385  4. Gratitude: 0.7095697  5. Love and care: 0.6902959  6. Pamper: 0.63285655  7. Financial aid: 0.6040691  8. Fraternity: 0.5798244  9. Friendship: 0.5769097  10. Compassion: 0.569711 | Concern | 1. Care: 1.0  2. Emphasize: 0.64810646  3. Concerned:0.62803364  4. Attention: 0.62540585  5. Thanks: 0.6242875  6. Weight: 0.61940664  7. Love and care: 0.5813702  8. Mind: 0.57272214  9. Cherish: 0.55067384  10. Respect and Love: 0.53444374 |
| Support | 1. Help: 1.0  2. Poverty alleviation: 0.77150357  3. Twinning: 0.76313967  4. Financial aid: 0.6714146  5. Poor households: 0.6660954  6. Difficult workers: 0.6521879  7. Poverty reduction: 0.6449658  8. Help for the needy: 0.63128424  9. Assistance: 0.6144574  10. Mutual help: 0.61011976 | Help | 1. Assistance: 1.0  2. Assist: 0.7722573  3. Substitute: 0.69083077  4. Please: 0.6769259  5. Door-to-door: 0.64940196  6. Expense: 0.647715  7. Begging: 0.6311947  8. Kindness: 0.62154  9. Self-reported: 0.60535645  10. Running around: 0.5939362 |
| Low-income insurance | 1. Low insurance: 1.0  2. Low-income households: 0.6686276  3. Five insurance: 0.6507863  4. Low income: 0.6496671  5. Exceptional hardship case: 0.62893414  6. Cooperative medical care: 0.6212828  7. Low-income pension: 0.6205236  8. Social security: 0.61298853  9. Urban residents: 0.59795034  10. Rural residents: 0.58358544 | Public Welfare | 1. Public Benefit: 1.0  2. Charity: 0.8390283  3. Public welfare activities: 0.757499  4. Public charity: 0.70489264  5. Commonweal: 0.6545077  6. Financial aid: 0.6489075  7. Philanthropy: 0.6481215  8. Volunteer: 0.64105225  9. NGO: 0.5964699  10. Outreach: 0.57096463 |
| Charity | 1. Charity: 1.0  2. Public benefit: 0.8390283  3. Philanthropy: 0.706045  4. Fundraising: 0.6979194  5. Public welfare activities: 0.6828167  6. Charities: 0.679218  7. Public Welfare: 0.66069627  8. Fundraising: 0.65902984  9. Crowdfunding: 0.65356916  10. Oxfam: 0.6098739 | Community Services | 1. Community Service: 1.0  2. Community activities: 0.67066073  3. Service items: 0.6020316  4. Social work: 0.5847992  5. Vocational training: 0.5714055  6. Social activities: 0.5708828  7. Lifelong education: 0.56054664  8. Adult education: 0.5305037  9. Outreach: 0.5227422  10. Neighborhood: 0.22638237 |
| Warmth | 1. Warmth: 1.0  2. Cool: 0.7091902  3. Pure and simple: 0.65550196  4. Refreshing: 0.65124136  5. Serenity: 0.6464978  6. Sadness: 0.64532113  7. Tenderness: 0.6436911  8. Fragrance: 0.63768655  9. Warm: 0.6370668  10. Genial: 0.6289383 | Light up | 1. Light up: 1.0  2. Illumination: 0.82462835  3. Release: 0.6896915  4. Bloom: 0.6436917  5. Flicker: 0.6229586  6. Dance: 0.572318  7. Candlelight: 0.54203695  8. Stand up: 0.53381157  9. Flash: 0.5272295  10. Flow of light: 0.5146159 |
| Virtues | 1. Virtues: 1.0  2. Character: 0.7207856  3. Traditional virtues: 0.6986904  4. Noble: 0.68950105  5. Sentimentality: 0.68400526  6. Quality: 0.6560137  7. Loyalty: 0.6341965  8. Noble sentiment: 0.6339355  9. Morality: 0.63013583  10. Helping people: 0.62732595 | Social Worker | 1. Social Worker: 1.0  2. Volunteer: 0.7069009  3. Social workers: 0.69864273  4. Social work: 0.6815002  5. Medical care: 0.6422986  6. Nursing staff: 0.6119826  7. Tutor: 0.6113929  8. Social welfare: 0.5859838  9. Caritas: 0.5674321  10. Nursing: 0.54012144 |
